# Using COVID-19 Vaccine Attitudes Found in Tweets to Predict Vaccine Perceptions in Traditional Surveys: Infodemiology Study

**DOI:** 10.2196/43700

**Published:** 2023-11-30

**Authors:** Nekabari Sigalo, Vanessa Frias-Martinez

**Affiliations:** 1 College of Information Studies University of Maryland College Park, MD United States

**Keywords:** social media, Twitter, COVID-19, vaccine, surveys, SARS-CoV-2, vaccinations, hesitancy

## Abstract

**Background:**

Traditionally, surveys are conducted to answer questions related to public health but can be costly to execute. However, the information that researchers aim to extract from surveys could potentially be retrieved from social media, which possesses data that are highly accessible and lower in cost to collect.

**Objective:**

This study aims to evaluate whether attitudes toward COVID-19 vaccines collected from the Household Pulse Survey (HPS) could be predicted using attitudes extracted from Twitter (subsequently rebranded X). Ultimately, this study aimed to determine whether Twitter can provide us with similar information to that observed in traditional surveys or whether saving money comes at the cost of losing rich data.

**Methods:**

COVID-19 vaccine attitudes were extracted from the HPS conducted between January 6 and May 25, 2021. Twitter’s streaming application programming interface was used to collect COVID-19 vaccine tweets during the same period. A sentiment and emotion analysis of tweets was conducted to examine attitudes toward the COVID-19 vaccine on Twitter. Generalized linear models and generalized linear mixed models were used to evaluate the ability of COVID-19 vaccine attitudes on Twitter to predict vaccine attitudes in the HPS.

**Results:**

The results revealed that vaccine perceptions expressed on Twitter performed well in predicting vaccine perceptions in the survey.

**Conclusions:**

These findings suggest that the information researchers aim to extract from surveys could potentially also be retrieved from a more accessible data source, such as Twitter. Leveraging Twitter data alongside traditional surveys can provide a more comprehensive and nuanced understanding of COVID-19 vaccine perceptions, facilitating evidence-based decision-making and tailored public health strategies.

## Introduction

### Background

The implementation of successful COVID-19 vaccine rollouts is essential for COVID-19 to remain under control globally. Although vaccines are essential in the global battle against COVID-19, vaccine hesitancy continues to be a barrier to effective and consistent vaccine rollout programs. According to the US Census Bureau’s Household Pulse Survey (HPS), individuals who reported being hesitant about receiving a COVID-19 vaccine cited concerns about side effects, safety, and a lack of trust in the vaccine or the government [1]. Although the number of vaccine-hesitant individuals continues to decline, the fact that vaccine hesitancy still exists interferes with infection control through vaccination.

Vaccine hesitancy has been fueled in part by the spread of vaccine misinformation, both in the media and on the web. In fact, the COVID-19 vaccine became a popular topic of discussion among social media users, with many individuals expressing their concerns about taking the vaccine on social media platforms [2]. Amid the new normal of self-quarantine and lockdown, Twitter (subsequently rebranded X) quickly emerged as an important means of COVID-19 communications and discussion [3]. This is in part due to the real-time availability of social media messaging, compared with traditional news reporting methods [4,5]. Twitter users often not only take to the platform to announce their own experiences and opinions about the pandemic but also see Twitter as a source of up-to-date information about the pandemic [6].

The COVID-19 vaccine conversation on social media platforms has been both beneficial and detrimental to vaccination efforts across the world [7]. Although the exact effect of social media on this unprecedented pandemic is difficult to quantify, there has been a constant battle between facts and misinformation, trust and fearmongering, and hope and anger [8]. Research has shown that social media use plays a role in the low acceptance of vaccines [9,10]. Therefore, studying the public COVID-19 vaccine–related discussion on social media can help researchers better understand attitudes related to the vaccine [9].

Traditionally, surveys are conducted to understand attitudes related to public health. For example, many studies leveraged surveys to examine COVID-19 vaccine hesitancy and compliance. In April 2020, Ward et al [11] administered 4 web-based, nationally representative surveys to adults in France to identify the reasons why individuals would or would not take the COVID-19 vaccine once it became available. Researchers found that nearly a quarter of the respondents refused to take the COVID-19 vaccine once it was made available to them, citing reasons such as not trusting vaccines in general; concerns about the expedited vaccine development process; and a lack of fear of COVID-19, deeming the vaccine unnecessary.

In the study by Wang et al [12], a cross-sectional, self-administered survey was conducted to evaluate the COVID-19 vaccine intent among nurses in Hong Kong, China. Researchers found higher rates of vaccine hesitancy compared with vaccine acceptance, with nurses citing concerns about the safety and efficacy of the vaccines. A web-based survey conducted in the study by Ruiz and Bell [13] attempted to identify the predictors of the intent to vaccinate against COVID-19 among Americans. Nearly 40% of the survey respondents refused to be vaccinated against COVID-19. Among the survey respondents, male, older, White, and married individuals and individuals with higher socioeconomic status were more likely to be vaccinated against COVID-19. Researchers also found that Republicans and Fox News viewers were less likely to get vaccinated, whereas individuals who were previously vaccinated for influenza were more likely to be vaccinated for COVID-19.

Although several studies have examined COVID-19 vaccine attitudes through surveys, to our knowledge, no study has evaluated the ability of Twitter, a newer data source, to predict the attitudes reflected in traditionally collected surveys, such as the HPS. In recent years, researchers have looked at social media as a data source, citing the availability of more readily available data and no- or low-cost data collection efforts [14,15]. Traditional mail, field, and telephone interviewer surveys come with high costs of administration, and even though web-based surveys eliminate the costs of postage, paper, printing, and data entry, the newer web-based survey services may still cost up to thousands of dollars for 1 survey [16]. Although relatively inexpensive compared with traditional surveys, web-based surveys are not always cost-effective [16]. Evaluating the ability of information extracted from social media to predict information found in traditional surveys would suggest whether researchers may use this more cost-effective data source to provide similar rich information to that seen in traditional surveys or whether saving money comes at the cost of losing rich data.

### Study Overview

The main objective of this study was to examine whether aggregate attitudes extracted from social media can predict vaccine attitudes collected via surveys. We hypothesized that social media may contain attitudes similar to those found in traditional surveys, with the added benefits of more readily available data and no- or low-cost data collection efforts. Predictive models of vaccine attitudes at the metropolitan level can be useful for 2 purposes. First, predictions can be used to identify metropolitan areas where vaccine hesitancy is high and create targeted campaigns to increase vaccination. Second, the relationships between sentiments and emotions and vaccine attitudes can be used to understand human perceptions of vaccines and create effective social media messages for vaccination campaigns. Specifically, we hypothesize that there is a direct, positive relationship between (1) positive sentiments and emotions found in Twitter data and the HPS and (2) negative sentiments and emotions found in Twitter data and the HPS survey.

## Methods

### Data Collection and Preprocessing

#### HPS Data

In April 2020, the US Census Bureau began releasing a cross-sectional nationally representative survey, the HPS, in an effort to assess the social and economic impacts of the COVID-19 pandemic on American households [17]. The data from this survey were made publicly available in near real time with the purpose of informing federal and state response and recovery planning [18]. The HPS sample was selected using a stratified random sampling method [19]. Data were collected via computer-assisted telephone interviewing, a data collection method in which surveyors use computer software to conduct telephone interviews with respondents [19].

On January 6, 2021, the US Census Bureau added COVID-19 vaccine–related questions to the HPS with the goal of understanding the factors contributing to vaccine hesitancy and compliance among Americans [20] (Table 1). These questions assessed COVID-19 vaccine receipt, whether respondents received or planned to receive all required doses, intentions to get vaccinated, and reasons why respondents refused to get vaccinated.

**Table 1:** COVID-19–related Household Pulse Survey questions.

Condition	Question	Responses
Age >18 years	Have you received a COVID-19 vaccine?	(1) Yes (2) No
Answered “yes” to “have you received a COVID-19 vaccine?”	Did you receive (or do you plan to receive) all required doses?	(1) Yes (2) No
Answered “no” to “have you received a COVID-19 vaccine?”	Once a vaccine to prevent COVID-19 is available to you, would you...	(1) Definitely get a vaccine (2) Probably get a vaccine (3) Be unsure about getting a vaccine (4) Probably not get a vaccine (5) Definitely not get a vaccine

Measures of vaccine compliance and hesitancy were assessed for each survey wave overall and in the metropolitan areas in Textbox 1. At the start of the survey period, January 2021, vaccine rollout in the United States had just begun, and with most people unvaccinated at that point, the intent to vaccinate was the only option. For the purposes of this analysis, individuals who answered that they would “definitely get a vaccine” or “probably get a vaccine” once available were considered *vaccine compliant*, and individuals who answered that they would “probably not get a vaccine,” or “definitely not get a vaccine” once available were considered *vaccine hesitant*.

The HPS refers to the data collection cycles as *weeks* for consistency with earlier phases, even though the cycles actually span a 2-week collection period. For this study, we used the HPS microdata from weeks 22 to 30, which were collected between January 6 and May 25, 2021, with response rates ranging from 6.4% to 7.5% (Table 2).

**Targeted metropolitan areas for data collection (January to May 2021).**

- New York–Newark–Jersey City, New York–New Jersey–Pennsylvania metropolitan area
- Los Angeles–Long Beach–Anaheim, California metropolitan area
- Chicago-Naperville-Elgin, Illinois-Indiana-Wisconsin metropolitan area
- Dallas–Fort Worth–Arlington, Texas metropolitan area
- Houston–The Woodlands–Sugar Land, Texas metropolitan area
- Washington-Arlington-Alexandria, District of Columbia–Virginia–Maryland–West Virginia metropolitan area
- Miami–Fort Lauderdale–Pompano Beach, FL metropolitan area
- Philadelphia-Camden-Wilmington, Pennsylvania–New Jersey–Delaware–Maryland metropolitan area
- Atlanta–Sandy Springs–Alpharetta, Georgia metropolitan area
- Phoenix-Mesa-Chandler, Arizona metropolitan area
- Boston-Cambridge-Newton, Massachusetts–New Hampshire metropolitan area
- San Francisco–Oakland–Berkeley, California metropolitan area
- Detroit-Warren-Dearborn, Michigan metropolitan area
- Seattle-Tacoma-Bellevue, Washington metropolitan area

**Table 2:** Household Pulse Survey data collection schedule.

Collection dates	Week	Response rate (%)
January 6-January 18, 2021	22	6.4
January 20-February 1, 2021	23	7.5
February 3-February 15, 2021	24	7.3
February 17-March 1, 2021	25	7.3
March 3-March 15, 2021	26	7.4
March 17-March 29, 2021	27	7.2
April 14-April 26, 2021	28	6.6
April 28-May 10, 2021	29	7.4
May 12-May 24, 2021	30	6.8

#### Twitter Data

To align with the HPS data collection period outlined in Table 2, the Twitter Streaming application programming interface, which provides access to a random sample of 1% of publicly available tweets, was used to collect tweets from the metropolitan areas represented in the HPS (Textbox 1) from January to May 2021. All tweets had *place* information (usually city and state). The place information found in tweets was used to determine the metropolitan area associated with each tweet. Next, to extract tweets related to COVID-19 vaccines, tweets were further filtered by matching variations of vaccine-related keywords, such as *vaccine, pfizer, moderna, johnson & johnson,* and *dose*. The tweet sample was further preprocessed to minimize *noise* resulting from tweets that matched our vaccine-related keywords but did not necessarily reflect the thoughts and opinions of individual Twitter users. For example, companies often promote job postings and advertisements on Twitter using targeted hashtags in hopes of reaching their target audience. To prevent these tweets from adding noise to the sample, tweets related to job postings and advertisements were removed by excluding tweets with hashtags and keywords such as *#jobs*, *#hiring*, and *#ad*.

#### Sentiment and Emotion Analysis of Tweets

To capture the attitudes found in COVID-19 vaccine–related tweets, a sentiment and emotion analysis of all tweets was conducted using the Natural Language Understanding Research Consortium (NRC) lexicon from the *Syuzhet* package in R (R Core Team) [21]. The NRC lexicon, developed by Saif Mohammad, contains a list of manually labeled English words and their associations with negative and positive sentiments and common human emotions, such as trust, fear, sadness, surprise, and disgust [22]. The *Syuzhet* package applies the NRC lexicon by independently evaluating and rating each word or expression within a tweet [23]. The *get_nrc_sentiment* function was applied to all tweets to calculate the valence of 8 different emotions (fear, joy, anticipation, anger, disgust, sadness, surprise, and trust), along with the overall positive and negative sentiments, toward the COVID-19 vaccine. To assess the accuracy of the sentiment classifier, a random sample of 1000 tweets was selected for manual classification as having a positive or negative sentiment. Among the 1000 tweets in the random sample, 734 (73.4%) were accurately classified by the automated sentiment classifier.

The percentage of tweets expressing the 8 emotions, along with the percentage of tweets expressing a positive or negative sentiment, was calculated at the metropolitan level. For the purposes of this analysis, we used the proportion of tweets with a positive sentiment and positive emotions toward vaccines as a proxy to capture vaccine compliance among Twitter users, and the proportion of tweets with a negative sentiment and negative emotions toward vaccines was used as a proxy to capture vaccine hesitancy among Twitter users.

### Data Analysis

Statistical analysis was conducted using the R software packages *betareg* and *GLMMadaptive* [24,25]. To determine whether COVID-19 vaccine attitudes on Twitter can predict the proportion of COVID-19 vaccine perceptions ultimately expressed in the HPS (unweighted), both generalized linear models (GLMs) and generalized linear mixed models (GLMMs) were constructed (Table 3). The models were developed using a total of 126 data points, including proportional vaccine compliance and hesitancy proxies from 14 metropolitan areas across 9 survey waves.

**Table 3:** Regression models evaluating the relationship between Twitter sentiments and emotions and HPS^a^ vaccine hesitancy and compliance.

Model	Features	Outcome
Model 1a (GLM^b^)	Percentage of positive sentiment Percentage of joy Percentage of surprise Percentage of trust Percentage of anticipation Percentage of survey week (fixed effect) Percentage of metropolitan area (fixed effect)	Percentage of vaccine-compliant HPS respondents
Model 1b (GLMM^c^)	Percentage of positive sentiment Percentage of joy Percentage of surprise Percentage of trust Percentage of anticipation Percentage of percentage of survey week (fixed effect) Percentage of metropolitan area (random effect)	Percentage of vaccine-compliant HPS respondents
Model 2a (GLM)	Percentage of negative sentiment Percentage of anger Percentage of disgust Percentage of sadness Percentage of fear Percentage of anticipation Survey week (fixed effect) Metropolitan area (fixed effect)	Percentage of vaccine-hesitant HPS respondents
Model 2b (GLMM)	Percentage of negative sentiment Percentage of anger Percentage of disgust Percentage of sadness Percentage of fear Percentage of anticipation Survey week (fixed effect) Metropolitan area (random effect)	Percentage of vaccine-hesitant HPS respondents

^a^HPS: Household Pulse Survey.

^b^GLM: generalized linear model.

^c^GLMM: generalized linear mixed model.

GLMs were implemented with both time and geographic location as fixed effects, whereas the GLMMs were a multilevel approach with time as a fixed effect and metropolitan area as a random effect. The main reason behind this dual modeling choice is that random effects can capture the latent variation in the data that cannot be explained by fixed effects or the error term [26]. Random effects represent factors with multiple levels, such as geographic location, and possess distinct components that vary across these levels. Random effects prove especially valuable when dealing with hierarchical or nested data structures, where observations are not independent but grouped at a higher level, and enable us to account for similarities within these groups and prevent overfitting [25]. However, if there is limited variability across locations, including location as a random effect may lead to unstable estimates or unreliable inferences [27]. In such cases, it is often better to treat location as a fixed effect or aggregate the data at a higher level. Thus, we constructed both GLMs and GLMMs to assess the prediction power of using metropolitan areas as either fixed or random effects. We fit beta regression models with the logit link, which is the most appropriate for modeling proportional data [28]. In beta regression, the outcome variable is assumed to follow a beta distribution. Prior to evaluating the models, we conducted assumption checks and checked for multicollinearity and outliers to determine whether the necessary conditions were met. These model diagnostics are presented in Multimedia Appendix 1.

As shown in Table 3, we constructed 2 models to predict vaccine compliance (models 1a, GLM, and 1b, GLMM) and 2 models to predict vaccine hesitancy (models 2a, GLM, and 2b, GLMM). In model 1a, we fit a GLM in which the predictor variables were each of the 5 positive Twitter-derived sentiment and emotion features and the outcome variable was the proportion of vaccine-compliant HPS respondents. This model controlled for survey week (time) and metropolitan area as fixed effects. In model 1b, to account for variations in time and location, we fit a GLMM with each of the 5 positive Twitter-derived sentiment and emotion features and survey week (time) as fixed effects and metropolitan area as a random effect.

By contrast, in model 2a, we fit a GLM in which the predictor variables were each of the 6 negative Twitter-derived sentiment and emotion features and the outcome variable was the proportion of vaccine-hesitant HPS respondents. This model controlled for survey week (time) and metropolitan area as fixed effects. In model 2b, we fit a GLMM with each of the 6 negative Twitter-derived sentiment and emotion features and survey week (time) as fixed effects and metropolitan area as a random effect. As anticipation can be perceived as both positive and negative, this emotion was included as a feature in all models.

### Ethical Considerations

This project does not meet the definition of human participant research under the purview of the University of Maryland Institutional Review Board according to federal regulations, section 45CFR46.102(e) [29].

## Results

### Descriptive Statistics

There were a total of 92,453 tweets from 32,645 users across the 14 metropolitan areas in this study (Table 4). The Los Angeles–Long Beach–Anaheim metropolitan area had the largest representation of tweets (21,500/92,453, 23.26%), whereas the New York–Newark–New Jersey metropolitan area had the largest representation of users (18,400/32,645, 56.36%). The maximum number of tweets by a single individual was 274 (from a user in the New York–Newark–New Jersey metropolitan area). There were a total of 240,242 respondents to the HPS across the 14 metropolitan areas and 9 waves in this study, with the largest sample being the sample from the Washington-Arlington-Alexandria metropolitan area (Table 5).

**Table 4:** Number of tweets (N=92,453) and users (N=32,645) by metropolitan area (January to May 2021).

Metropolitan area, state	Tweets, n (%)	Users, n (%)	Weekly number of tweets, mean (SD)	Weekly number of users, mean (SD)
Atlanta–Sandy Springs–Alpharetta, Georgia	4234 (4.58)	1542 (4.72)	470 (186)	254 (106)
Boston-Cambridge-Newton, Massachusetts–New Hampshire	3019 (3.27)	1298 (3.98)	335 (133)	218 (83)
Chicago-Naperville-Elgin, Illinois-Indiana-Wisconsin	5821 (6.3)	2561 (7.84)	647 (252)	426 (160)
Dallas–Fort Worth–Arlington, Texas	6203 (6.71)	2299 (7.04)	689 (265)	371 (133)
Detroit-Warren-Dearborn-Michigan	1082 (1.17)	518 (1.59)	120 (56)	84 (40)
Houston–The Woodlands–Sugar Land, Texas	5125 (5.54)	2421 (7.42)	569 (234)	388 (145)
Los Angeles–Long Beach–Anaheim, California	21,500 (23.26)	5429 (16.63)	2389 (983)	891 (344)
Miami–Fort Lauderdale–Pompano Beach, Florida	1954 (2.11)	849 (2.6)	217 (74)	131 (40)
New York–Newark–Jersey City, New York–New Jersey–Pennsylvania	18,400 (19.9)	7259 (22.24)	2044 (683)	1272 (400)
Philadelphia-Camden-Wilmington, Pennsylvania–New Jersey–Delaware-Maryland	3652 (3.95)	1406 (4.31)	406 (156)	250 (88)
Phoenix-Mesa-Chandler, Arizona	4778 (5.17)	1573 (4.82)	531 (183)	260 (81)
San Francisco–Oakland–Berkeley, California	6376 (6.9)	2008 (6.15)	708 (261)	347 (116)
Seattle-Tacoma-Bellevue, Washington	3089 (3.34)	1333 (4.08)	343 (157)	227 (103)
Washington-Arlington-Alexandria, District of Columbia–Virginia–Maryland–West Virginia	7220 (7.81)	2419 (7.41)	802 (313)	436 (155)

**Table 5:** Number of survey respondents (N=240,242) by city.

Metropolitan area, state	Respondents, n (%)	Weekly number of respondents, mean (SD)
Atlanta–Sandy Springs–Alpharetta, Georgia	12,611 (5.25)	1261 (48)
Boston-Cambridge-Newton, Massachusetts–New Hampshire	20,078 (8.36)	2008 (121)
Chicago-Naperville-Elgin, Illinois-Indiana-Wisconsin	16,044 (6.68)	1604 (89)
Dallas–Fort Worth–Arlington, Texas	15,859 (6.6)	1586 (88)
Detroit-Warren-Dearborn-Michigan	12,149 (5.06)	1215 (88)
Houston–The Woodlands–Sugar Land, Texas	14,179 (5.9)	1418 (125)
Los Angeles–Long Beach–Anaheim, California	17,006 (7.08)	1701 (101)
Miami–Fort Lauderdale–Pompano Beach, Florida	11,641 (4.85)	1164 (67)
New York–Newark–Jersey City, New York–New Jersey–Pennsylvania	19,730 (8.21)	1973 (124)
Philadelphia-Camden-Wilmington, Pennsylvania–New Jersey–Delaware–Maryland	20,240 (8.42)	2024 (162)
Phoenix-Mesa-Chandler, Arizona	14,027 (5.84)	1403 (106)
San Francisco–Oakland–Berkeley, California	17,787 (7.4)	1779 (78)
Seattle-Tacoma-Bellevue, Washington	18,615 (7.75)	1862 (106)
Washington-Arlington-Alexandria, District of Columbia–Virginia–Maryland–West Virginia	30,276 (12.6)	3028 (227)

### Attitudes Toward COVID-19 Vaccines in Twitter Data

A sentiment analysis classified most tweets (50,415/92,453, 54.53% of tweets overall) across all metropolitan areas as having a positive sentiment (Table 6). The Washington-Arlington-Alexandria metropolitan area had the largest proportion of tweets with a positive sentiment (53,715/92,453, 58.1%), whereas the Miami–Fort Lauderdale–Pompano Beach metropolitan area had the lowest proportion of tweets with a positive sentiment (47,059/92,453, 50.9%). Tweets with a negative sentiment held the smallest proportions across all metropolitan areas (13,970/92,453, 15.11% of tweets overall). The Los Angeles–Long Beach–Anaheim metropolitan area had the largest proportion of tweets with a negative sentiment (15,162/92,453, 16.4%), whereas the Miami–Fort Lauderdale–Pompano Beach metropolitan area had the lowest proportion of tweets with a negative sentiment (11,926/92,453, 12.9%).

**Table 6:** Distribution of sentiments and emotions found in COVID-19 vaccine tweets (N=92,453; January to May 2021).

Sentiment or emotion	Tweets, n (%)
Positive sentiment	50,415 (54.53)
Trust	41,317 (44.69)
Anticipation	32,127 (34.75)
Fear	27,227 (29.45)
Sadness	24,935 (26.97)
Joy	24,241 (26.22)
Anger	21,671 (23.44)
Surprise	20,562 (22.24)
Disgust	14,746 (15.95)
Negative	13,970 (15.11)

The emotion analysis revealed trust as the predominantly expressed emotion in COVID-19 vaccine tweets across all metropolitan areas (41,317/92,453, 44.69%). The most perceived negative emotion across all metropolitan areas was fear (27,227/92,453, 29.45%). The least perceived positive emotions were joy (24,241/92,453, 26.22%) and surprise (20,562/92,453, 22.24%), whereas the least perceived negative emotions were anger (21,671/92,453, 23.44%) and disgust (14,746/92,453, 15.95%). Examples of tweets expressing positive, neutral, and negative sentiments are presented in Textbox 2.

**Examples of tweets expressing a positive or negative sentiment toward COVID-19 vaccines.**

**Positive sentiments**

- “Feeling blessed to be healthy this birthday. My two biggest presents are coming in the next week: Inauguration and my second vaccine.”
- “Hubby received his first vaccine does this morning-the sense of relief is for real, folks. #vaccinated”
- “With my granddaughter Aurora, Andy, and Elliot. I can see them again and give them a hug now that I am fully Covid 19 vaccinated. I have had both shots plus over 2 weeks since shot two. Thank you President Biden.”
- “My mom gets her second dose Sunday, big relief!”
- “I love so much that I got vaccinated today.”
- “Proud to work for you @bswhealth-my parents received their COVID vaccines this week at BUMC and said it was so quick and easy and the staff were so friendly! Thank you for taking care of them.”

**Negative sentiments**

- “This is from the Pfizer v-a-c-c-i-n-e. Please understand these shots cause harm. Injury is REAL & not rare. It’s a shame these poor people are being gaslighted, & media giants are censoring them.”
- “They way my people been bugging me about this d*mn vaccine, I’m not getting that s*it.”
- “No way!! No more lockdowns!! No vaccines!!! Oh and if your so concerned about the virus how about no illegals!!! Thank goodness for New Hampshire and Florida!! Go out.”
- Clearly you are ignorant of the fact that they said even if you get the vaccine you still have to wear a mask, social distance & deal with all the same bull shit draconian orders. Even after blatant evidence you still want to get it. Heres 100% evidence of brain wash mind control.”
- “I am 80. You can have my vaccine. I refuse to get one. I take 2 grams of vitamin C hourly. That makes me IMMUNE. Read: Linus Pauling. No mask. I am out every day working & walking in the park. Paul Kangas 4 Governor.”
- “F the stupid vaccine.”

### Attitudes Toward COVID-19 Vaccines in the HPS Data

Most survey respondents (127,833/240,242, 53.21%) across all metropolitan areas indicated that they received a COVID-19 vaccine, ranging from 50.2% (7041/14,027) of the survey respondents in the Phoenix-Mesa-Chandler metropolitan area to 56.4% (10,032/17,787) of the survey respondents in the San Francisco–Oakland–Berkeley metropolitan area (Table 7). Among the respondents who indicated that they received a COVID-19 vaccine, the majority (65,195/127,833, 51%) also indicated that they received or planned to receive all required doses. Among the respondents who indicated that they had not received a COVID-19 vaccine, the majority (89,759/112,409, 79.85% combined) indicated that they *probably* or *definitely* would get vaccinated), ranging from 48% (6733/14,027) of the survey respondents in the Phoenix-Mesa-Chandler metropolitan area to 75.2% (13,376/17,787) of the survey respondents in the San Francisco–Oakland–Berkeley metropolitan area. For the purposes of this analysis, individuals who answered they that would “definitely get a vaccine” or “probably get a vaccine” once available were considered *vaccine compliant*, and individuals who answered that they would “probably not get a vaccine” or “definitely not get a vaccine” once available were considered *vaccine hesitant*.

**Table 7:** Distribution of survey responses (unweighted; January to May 2021).

Question and responses		Respondents, n (%)
**Have you received a COVID-19 vaccine? (N=** **240,242)**		
	Yes	127,833 (53.21)
	No or did not answer	112,409 (46.79)
**Did you receive (or do you plan to receive) all required doses? (n=127,833)**		
	Yes	65,195 (51)
	No or did not answer	62,638 (49)
**Once a vaccine to prevent COVID-19 is available to you, would you... (n=112,409)**		
	Definitely get a vaccine	69,233 (61.59)
	Probably get a vaccine	20,526 (18.26)
	Be unsure about getting a vaccine	3114 (2.77)
	Probably not get a vaccine	10,836 (9.64)
	Definitely not get a vaccine	8700 (7.74)

### Predicting HPS Vaccine Attitudes Using Twitter-Based Attitudes

We evaluated the performance of each GLM in terms of R-squared value and root mean square error (RMSE). Model 1a revealed significant associations (*P*<.001) between the percentage of vaccine-compliant HPS respondents and the percentage of tweets expressing a positive sentiment and trust (Table 8). The R-squared value for the vaccine-compliant GLM (model 1a) was 94.11%, and the RMSE was 0.053, which suggests that we can predict vaccine compliance in the HPS fairly well using positive sentiments and emotions found on Twitter. The GLM coefficients showed that an increase in the percentage of tweets expressing a positive sentiment (*P*<.001) was significantly associated with an increase in the percentage of vaccine-compliant HPS respondents. By contrast, an increase in the percentage of tweets expressing trust (*P*<.001) was significantly associated with a decrease in the percentage of vaccine-compliant HPS respondents.

**Table 8:** Model results.

Model and features			β coefficient (SE)		*P* value		R-squared (%)			Root mean square error	
**Model 1a (GLM^a^)**								94.1			0.053
	Percentage of positive sentiment	5.007 (.865)		<.001^b^							
	Percentage of joy	.043 (1.482)		.98							
	Percentage of surprise	1.084 (1.163)		.35							
	Percentage of trust	−4.696 (.865)		<.001^b^							
	Percentage of anticipation	.930 (1.197)		.44							
**Model 1b (GLMM^c^)**								82.5			0.062
	Percentage of positive sentiment	4.791 (.865)		<.001^b^							
	Percentage of joy	.271 (1.483)		.86							
	Percentage of surprise	.942 (1.157)		.42							
	Percentage of trust	−4.529 (.906)		<.001^b^							
	Percentage of anticipation	1.239 (1.107)		.26							
**Model 2a (GLM)**								93.2			0.01
	Percentage of negative sentiment	−1.340 (.581)		.02^b^							
	Percentage of anger	.382 (.556)		.49							
	Percentage of disgust	−.356 (.612)		.56							
	Percentage of sadness	−1.011 (.625)		.11							
	Percentage of fear	.715 (.532)		.18							
	Percentage of anticipation	−.382 (.369)		.30							
**Model 2b (GLMM)**								9.4			0.032
	Percentage of negative sentiment	−1.334 (.617)		.03^b^							
	Percentage of anger	.425 (.587)		.47							
	Percentage of disgust	−.312 (.657)		.64							
	Percentage of sadness	−1.015 (.662)		.13							
	Percentage of fear	.643 (.567)		.26							
	Percentage of anticipation	−.456 (.388)		.24							

^a^GLM: generalized linear model.

^b^Statistically significant results (α=.05).

^c^GLMM: generalized linear mixed model.

When we compared the vaccine-compliant GLM (model 1a) with the vaccine-compliant GLMM with metropolitan area as a random effect (model 1b), we observed a lower R-squared value (82.5%) and higher RMSE (0.062). Model 1b results also showed that an increase in the percentage of tweets expressing a positive sentiment (*P*<.001) was significantly associated with an increase in the percentage of vaccine-compliant HPS respondents. By contrast, an increase in the percentage of tweets expressing trust (*P*<.001) was significantly associated with a decrease in the percentage of vaccine-compliant HPS respondents.

Model 2a revealed significant associations (*P*<.05) between the percentage of vaccine-hesitant HPS respondents and the percentage of tweets expressing a negative sentiment. The R-squared value for the vaccine-hesitant GLM (model 2a) was similar to that of the vaccine-compliant GLM (93.17%). However, the vaccine-hesitant GLMM showed a much lower R-squared value (9.4%) and slightly higher RMSE (0.032). When compared with the vaccine-hesitant GLM (model 2a), whose only difference from the GLMM was the use of metropolitan area as a random effect, these results revealed that metropolitan area, as a fixed effect, and negative tweet sentiment (statistically significant in both model 2a and 2b) contributed to the majority of the variation in the percentage of vaccine-hesitant HPS respondents. When looking at the regressors, the results of both the vaccine-hesitant models, model 2a and 2b, showed that an increase in the percentage of tweets expressing a negative sentiment (*P*=.02 and *P*=.03, respectively) was associated with a decrease in the percentage of vaccine-hesitant HPS respondents.

## Discussion

### Principal Findings

In this study, we sought to determine whether the sentiments and emotions found in COVID-19 vaccine tweets can predict the vaccine hesitancy and compliance expressed in the US Census Bureau’s HPS. Depending on the model, GLMs and GLMMs showed significant relationships between (1) the percentage of vaccine-compliant HPS respondents and percentage of tweets expressing a positive sentiment and trust and (2) the percentage of vaccine-hesitant HPS respondents and percentage of tweets expressing a negative sentiment. Positive perceptions expressed on Twitter performed well in predicting positive perceptions in the survey for both GLMs and GLMMs, whereas negative perceptions expressed on Twitter performed well in predicting negative perceptions in the survey only for the GLM.

### Study Findings in Context

The main objective of this study was to examine whether aggregate attitudes extracted from social media can predict vaccine attitudes collected via surveys. Specifically, we hypothesized that there is a direct, positive relationship between (1) positive sentiments found in Twitter and the HPS survey and (2) negative sentiments found in Twitter and the HPS survey. We expected to see a positive relationship between positive sentiments and emotions on Twitter and vaccine compliance in the HPS, as suggested in a previous study that showed a positive relationship between positive sentiment scores in COVID-19 vaccine–related tweets and an increase in vaccination rates [30]. The results of both vaccine-compliant models revealed, as expected, significant positive relationships between the percentage of vaccine-compliant HPS respondents and percentage of tweets expressing a positive sentiment. However, in both vaccine-compliant models, the direction of one of the statistically significant relationships that were revealed was not what we expected. Both vaccine-compliant models revealed a significant inverse relationship between the vaccine-compliant measure in the HPS and percentage of tweets expressing trust.

We also expected to see a positive relationship between negative sentiments and emotions on Twitter and vaccine hesitancy in the HPS. Although not significant, the percentages of tweets expressing anger and fear were, as expected, positively related to increases in vaccine hesitancy for both GLM and GLMM. However, the vaccine-hesitant GLM and GLMM both revealed a significant inverse relationship between the vaccine-hesitant measure in the HPS and percentage of tweets expressing a negative sentiment.

The unexpected direction of the relationship between some vaccine perceptions in the survey and vaccine perceptions found on Twitter might be explained via ecological fallacy. An ecological fallacy occurs when an inference is made about the relationship between variables at an aggregate or a group level (eg, the level of a neighborhood, city, or country) based solely on the relationship between the same variables at an individual level [31]. This can be problematic because the relationship between variables at the group level may differ from the relationship between the same variables at the individual level because of factors such as aggregation bias and other contextual factors.

Another possible explanation for the unexpected findings on some of the relationships between vaccine perceptions in the survey and vaccine perceptions found on Twitter could be the possibility that some individuals’ web-based personas may not match their reality. For example, an individual might be obligated to get a vaccine because of their job or an upcoming travel, making them vaccine compliant, but rant about it on the web. In our sample data, this type of person would be classified as *provaccine* instead of *antivaccine* in the HPS but would also contribute to the negative perceptions found on Twitter. These findings also align with prior research that suggested an individual’s web-based persona may differ from their offline identity [32-34]. This offline identity is often limited by physical, emotional, and financial circumstances that may be beyond an individual’s control [33,35-37]. However, individuals have complete control over the identity they choose to present on the web [32-34]. The inverse relationship between the vaccine-hesitant measure in the HPS and percentage of tweets expressing a negative sentiment may have also been due to the use of sarcasm in tweets, where the text itself contradicts what is actually meant by the user [38].

The findings of this study contribute to the literature in 2 ways. First, although many studies have examined COVID-19 vaccine acceptance by extracting information from either surveys or social media, to our knowledge, no study has evaluated the relationship between these vastly different data sources. Unlike social media data collection, surveys come with postage, paper, printing, interviewer, and data entry costs, making them costly to administer [39]. Evaluating the relationship between the attitudes found in surveys and those found on social media allows researchers to determine whether social media data can be trusted to reveal the same information that can be extracted from traditional surveys or whether there is a risk of losing important information in exchange for cutting costs. In this study, we found that COVID-19 vaccine attitudes in the HPS, measured as vaccine compliance and hesitancy, can be predicted using social media attitudes toward vaccines, measured via sentiments and emotions toward vaccines. The results of this study support the efforts of researchers, who over the past few years have looked at social media as a data source, citing the availability of readily available data and no- or low-cost data collection efforts [40,41].

This study makes further contributions by revealing the sentiments and emotions found in tweets across different metropolitan areas. This builds upon several other studies that leveraged natural language processing (NLP) methods, such as sentiment analysis, emotion analysis, and topic modeling, to examine vaccine-related perceptions [42-44]. In this study, we found that most tweets expressed a provaccine sentiment, across all metropolitan areas. However, many tweets also expressed negative feelings and anticipations. This supports previous work, where researchers found many discussions about vaccine hesitancy but ultimately found most tweets to have a positive sentiment [45]. This study also revealed trust as the dominant emotion found in tweets. This supports the results of a prior study that also found trust to be the dominant emotion expressed in tweets during an earlier period [46]. A comparison of these results shows that the vaccine conversation on Twitter remained relatively consistent over time.

Comparing COVID-19 vaccine perceptions on Twitter with attitudes in traditional public health surveys offers several benefits. Twitter serves as a platform for immediate and widespread dissemination of information. Analyzing vaccine perceptions on Twitter can help identify emerging issues or concerns related to COVID-19 vaccines at an early stage. This early detection allows public health authorities to address misconceptions, respond to emerging challenges, and promptly adapt their communication strategies. For example, the study results suggest that both models may be beneficial when deciding which cities to implement vaccine campaigns in, and the vaccine-compliant model can be used to better understand the role sentiments play in vaccination behaviors. This type of model can be used to craft effective social media messages related to COVID-19 vaccination.

Twitter provides a platform for a wide range of voices and opinions, including those of individuals with varying backgrounds, beliefs, and experiences. Comparing Twitter data with survey data allows for the exploration of diverse perspectives and can uncover viewpoints that may not be captured through traditional surveys alone. This broader range of perspectives enhances the understanding of the complexities surrounding vaccine perceptions. Twitter data also allow for the real-time monitoring of public sentiment and reactions toward COVID-19 vaccines. This timely information can provide valuable insights into evolving trends, emerging concerns, and the impact of specific events or interventions. By comparing Twitter discussions with survey responses, researchers can identify shifts in public opinion and monitor the effectiveness of public health communication strategies in real time.

Comparison with Twitter data can complement the findings of traditional surveys, providing a more comprehensive understanding of vaccine perceptions. Twitter data can provide contextual information, qualitative insights, and real-world examples that enrich the analysis of survey responses. The combination of both sources offers a more nuanced and holistic understanding of public attitudes toward COVID-19 vaccines.

This study provides further evidence for the benefits of using social media data for public health research. The overarching contribution of this work suggests the adoption of alternative data sources and NLP techniques to assist in public health decision-making.

### Limitations and Future Work

Considering the limitations of this study may lead to future, related work. This study emphasizes the use of Twitter as a data source, but the lack of representation among Twitter users leads to bias in the sample and contributes to sampling errors. For example, Twitter users tend to be younger, be more educated, have higher incomes, and be more liberal [47]. The lack of representation among Twitter users suggests the limited generalizability of the results to the larger population. Adding to this lack of representation is the limited sample of tweets available to the public via the Twitter Streaming application programming interface, which makes available a random sample of 1% of all tweets made by Twitter users at any given time [48]. In addition, in studies assessing COVID-19 vaccine perceptions using social media data, individuals who do not have access to social media are systematically excluded from the analysis sample.

The lack of demographic information on Twitter users is also a limitation to using Twitter as a data source. The absence of demographic information, such as age, gender, income, and education makes it challenging to understand the characteristics of the Twitter users who generate the data. This lack of information may lead to biased or incomplete analyses and limit the generalizability of the findings. In addition, the absence of demographic data makes it difficult to compare Twitter data with data from other sources that do contain demographic information, such as survey data. Despite these limitations, Twitter data can still be useful in certain contexts.

It should also be acknowledged that the HPS data are also subject to sampling errors due to sample design, nonresponse, weighting adjustments, and measurement errors [49]. As a result, the true relationship between aggregate attitudes extracted from social media and vaccine attitudes collected via surveys may be different from what was revealed in this study.

Future studies should endeavor to use other NLP approaches, such as topic modeling, to compare public perceptions of the COVID-19 vaccine on social media with those found in surveys. The survey used in this study, the HPS, presented respondents with in-depth questions related to why they were vaccine hesitant, so applying topic models to tweets may reveal some of the same attitudes and themes as those expressed in the survey. Future studies may also involve pulling data from other social media platforms, such as Facebook, and comparing the overall perceptions reflected across all media.

### Conclusions

The ongoing COVID-19 pandemic requires consistent monitoring and data-driven public health policies. To slow the spread of the virus, public health officials have stressed that vaccines are essential in the worldwide battle against COVID-19. However, vaccine hesitancy continues to be a barrier to effective and consistent vaccine rollout programs. Prior efforts have used surveys to gauge attitudes toward the COVID-19 vaccine, but this study suggests that these public perceptions may also be extracted from a readily available, low-cost data source, social media. In this study, we validated social media as a data source by evaluating the relationship between the attitudes expressed among Twitter users and attitudes expressed among respondents to the HPS as well as the ability of attitudes expressed among Twitter users to predict vaccine compliance and hesitancy among the HPS respondents. Leveraging Twitter data alongside traditional surveys can provide a more comprehensive and nuanced understanding of COVID-19 vaccine perceptions, facilitating evidence-based decision-making and tailored public health strategies.

## Appendix

Multimedia Appendix 1 Model diagnostics.

## Abbreviations

GLM: generalized linear model
GLMM: generalized linear mixed model
HPS: Household Pulse Survey
NLP: natural language processing
NRC: Natural Language Understanding Research Consortium
RMSE: root mean square error
