# Supplementary material for: Using COVID-19 Vaccine Attitudes Found in Tweets to Predict Vaccine Perceptions in Traditional Surveys: Infodemiology Study
Source: JMIR Infodemiology. 2023 Nov 30;3:e43700. doi: 10.2196/43700 (PMC10691448; doi:10.2196/43700)

**Multimedia Appendix 1.** Model Diagnostics**.**

## Diagnostic Plots

**Figure S1. Diagnostic Plots for Model 1a (GLM): % Vaccine Compliant HPS Respondents.** The diagnostic plots indicate the assumptions of beta regression have been met.
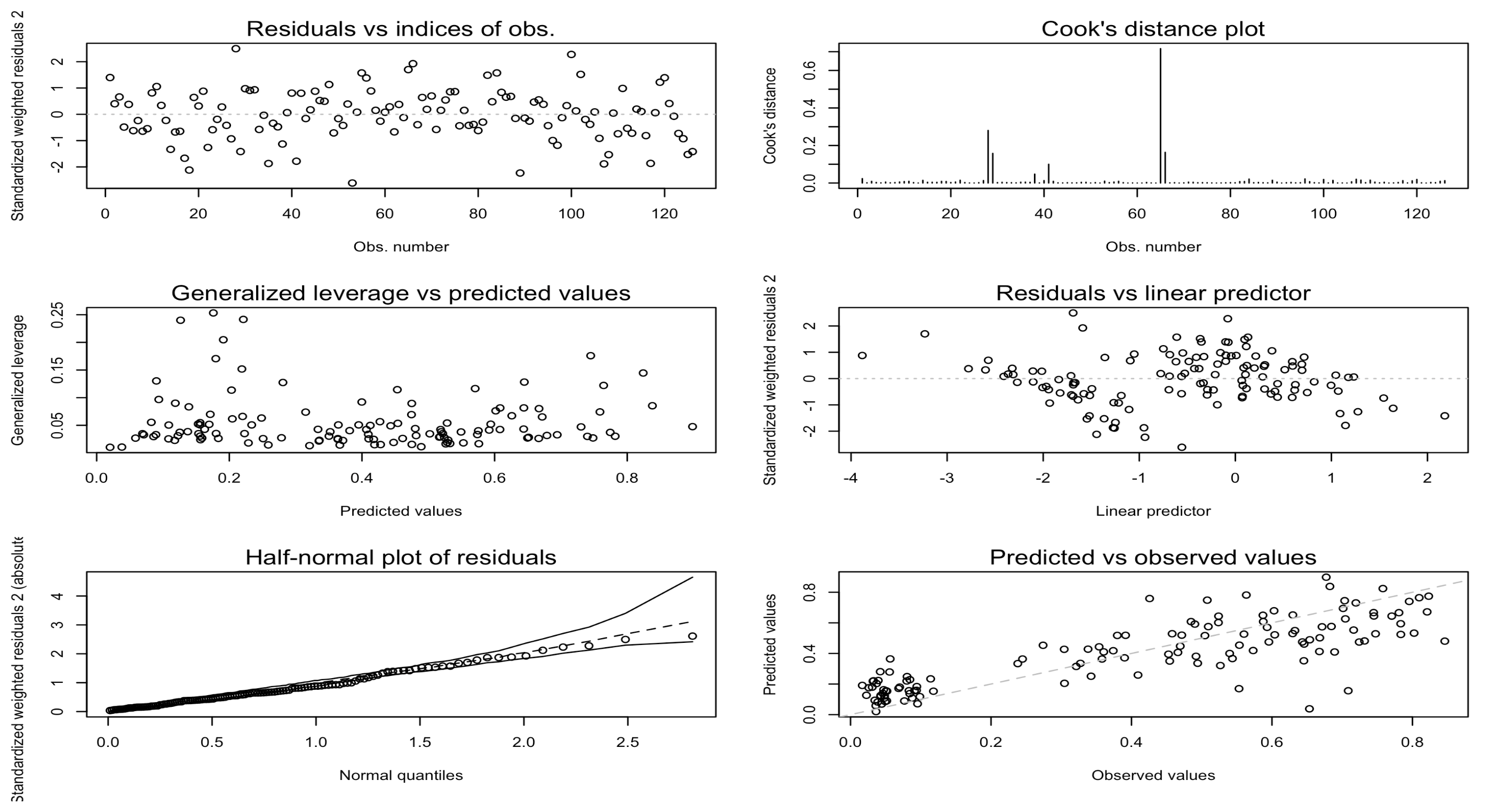


**Figure S2. Diagnostic Plots for Model 2a (GLM): % Vaccine Hesitant HPS Respondents.** The diagnostic plots indicate the assumptions of beta regression have been met.


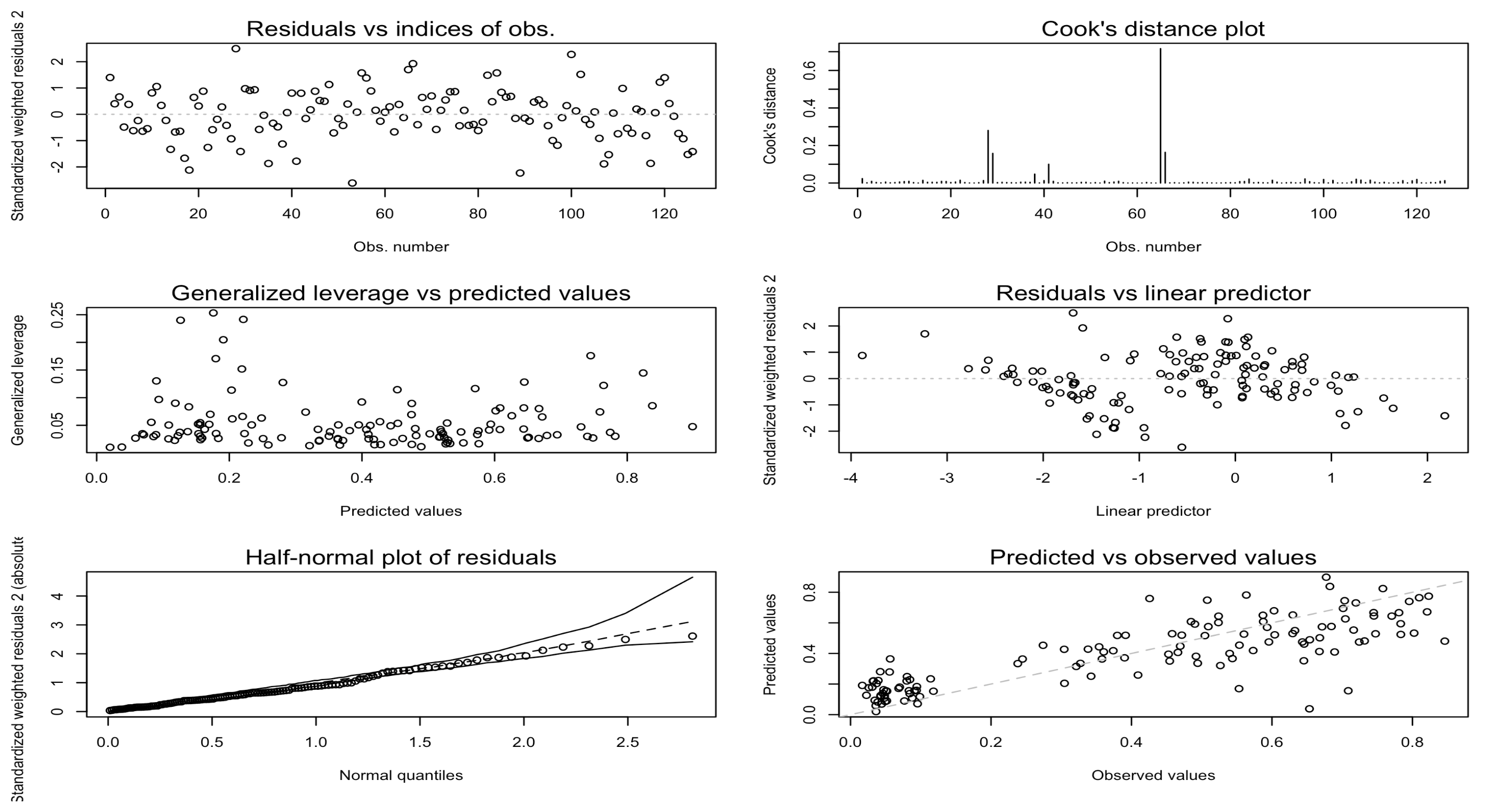


## Fitted vs Actuals

Each of the following plots serve as a visual representation to assess the performance of each model. These plots compare the predicted values generated by the model with the actual observed values from the dataset.

**Figure S3. Model 1a (GLM) Fitted vs Actuals: % Vaccine Compliant HPS Respondents**

**
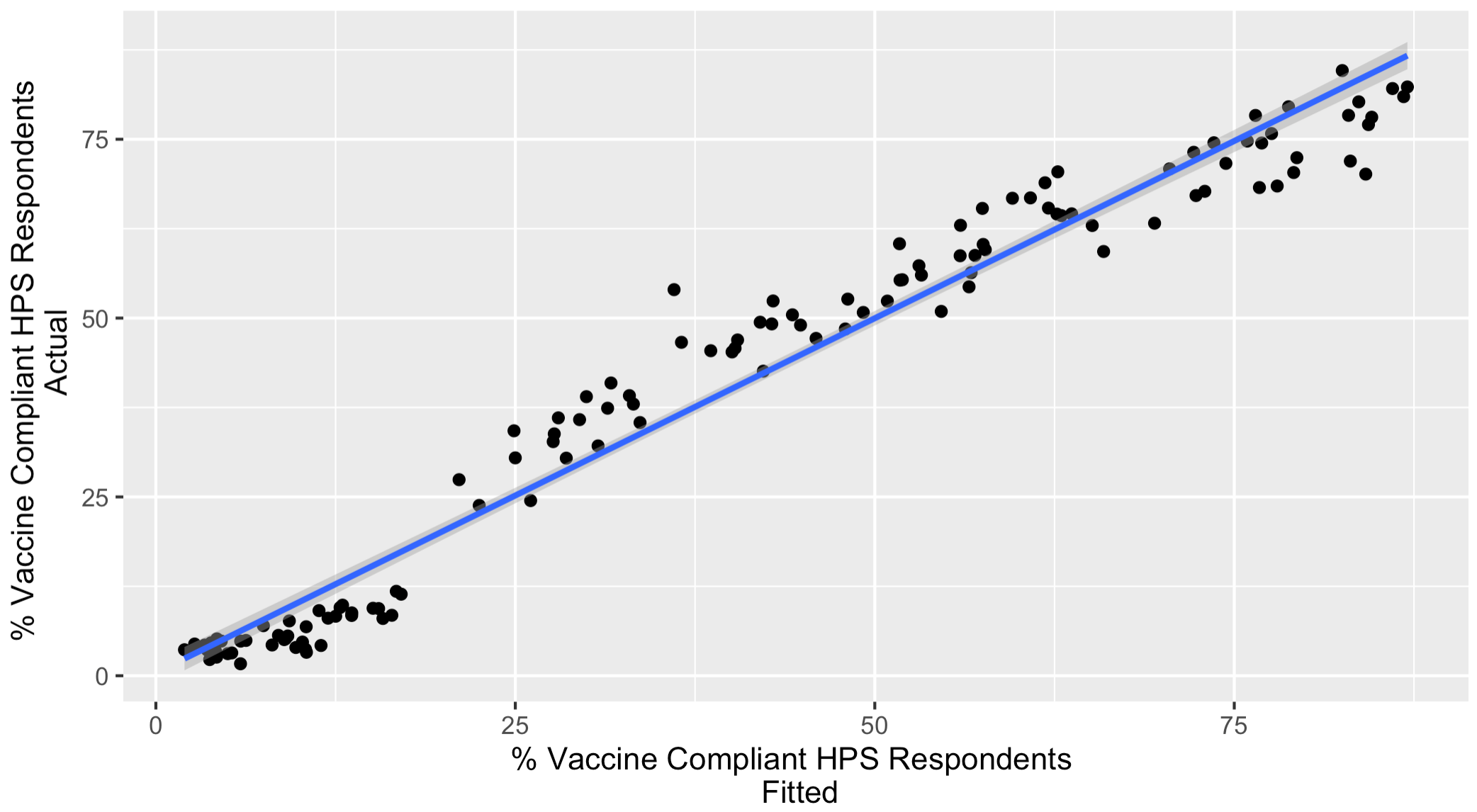
**

**Figure S4. Model 1b (GLMM) Fitted vs Actuals: % Vaccine Compliant HPS Respondents**

**
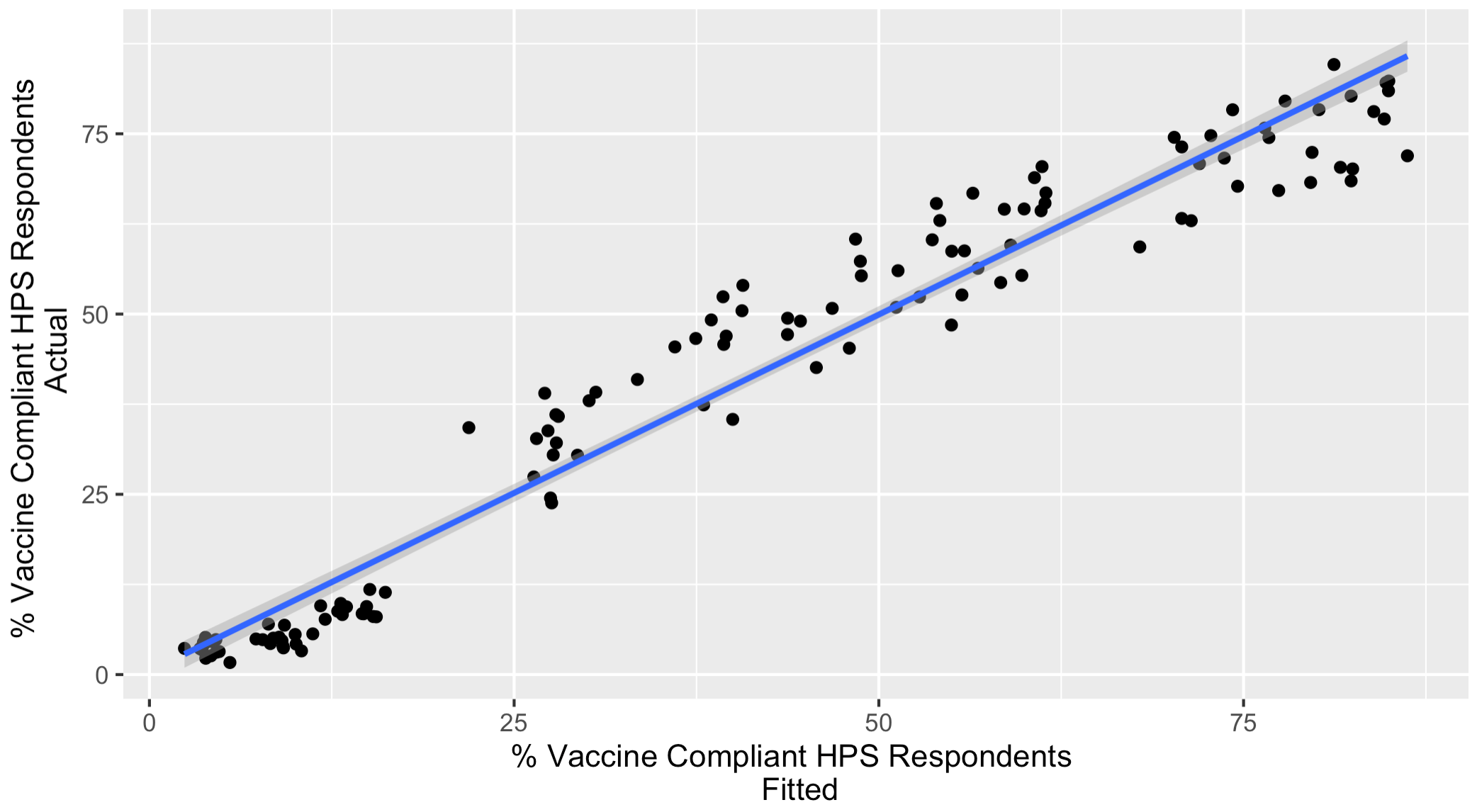
**

**Figure S5. Model 2a (GLM) Fitted vs Actuals: % Vaccine Hesitant HPS Respondents**

**
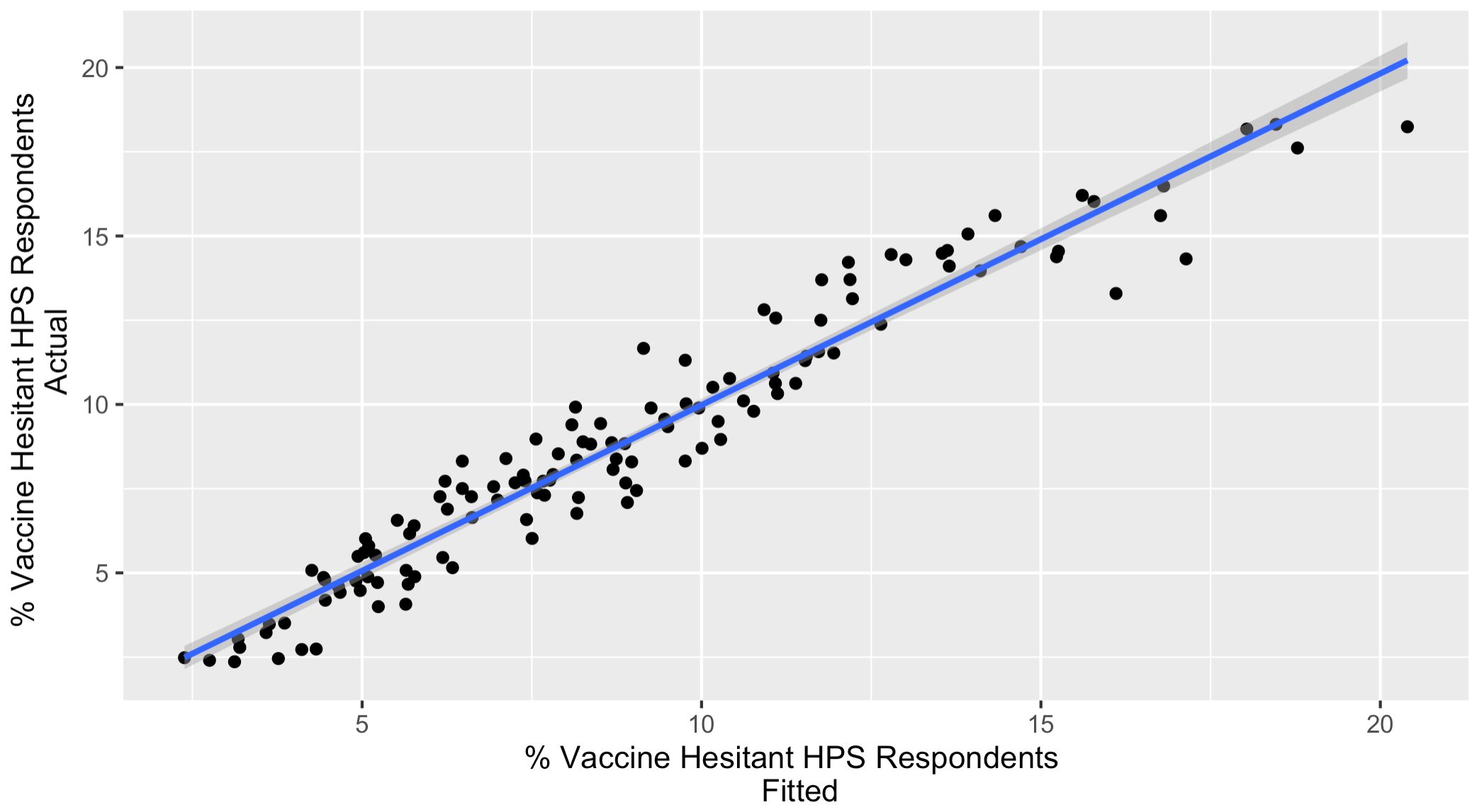
**

**Figure S6. Model 2b (GLMM) Fitted vs Actuals: % Vaccine Hesitant HPS Respondents**


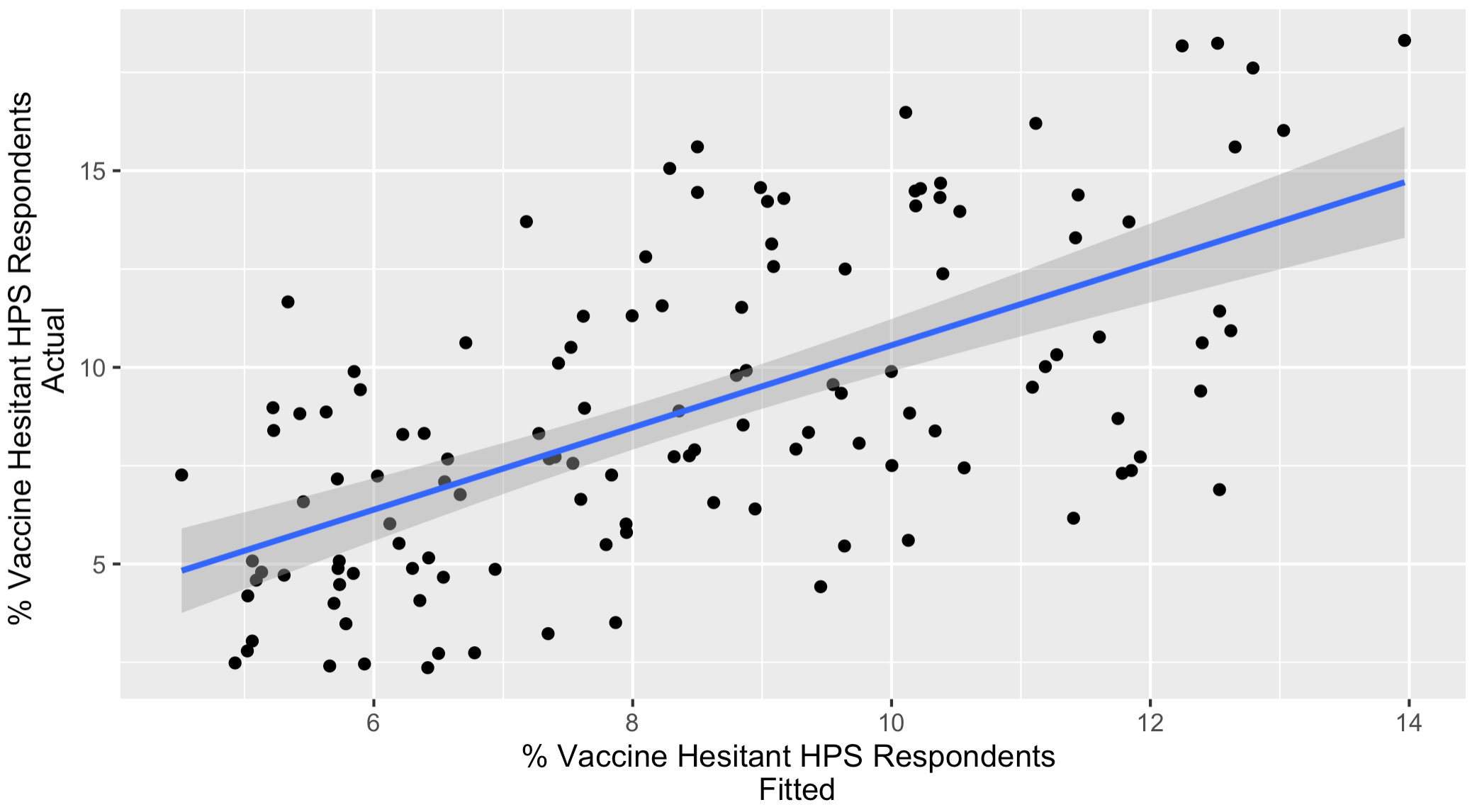


## Collinearity

**Table S1. Variance Inflation Factor (VIF) Assessing Multicollinearity.** We checked for collinearity between predictor variables using the Variance Inflation Factor (VIF). Using a threshold of less than 5, we determined there was no significant collinearity present.

| **Model** | **Variable** | **VIF** |
| --- | --- | --- |
| % Vaccine Compliant HPS Respondents | positive | 1.640602 |
|  | joy | 2.642618 |
|  | surprise | 1.365239 |
|  | trust | 1.367217 |
|  | anticipation | 2.034014 |
| % Vaccine Hesitant HPS Respondents | negative | 1.490458 |
|  | anger | 1.803852 |
|  | disgust | 1.568587 |
|  | sadness | 2.687337 |
|  | fear | 2.729726 |
|  | anticipation | 1.542728 |

## Predictors vs Outcomes

The following plots of survey estimates against each of the explanatory variables are shown to give an idea of variation and possible issues with influential data points. These plots also provide some insight into the model coefficients that appear to be in the “wrong” direction.

**Figure S7. Relationship Between Predictors and % Vaccine Compliant HPS Respondents.** The percentage of tweets expressing positive sentiment, joy, and anticipation have a positive relationship with the percent of vaccine compliant survey respondents. The percentage of tweets expressing surprise and trust have a weak negative relationship with the percent of vaccine compliant survey respondents.


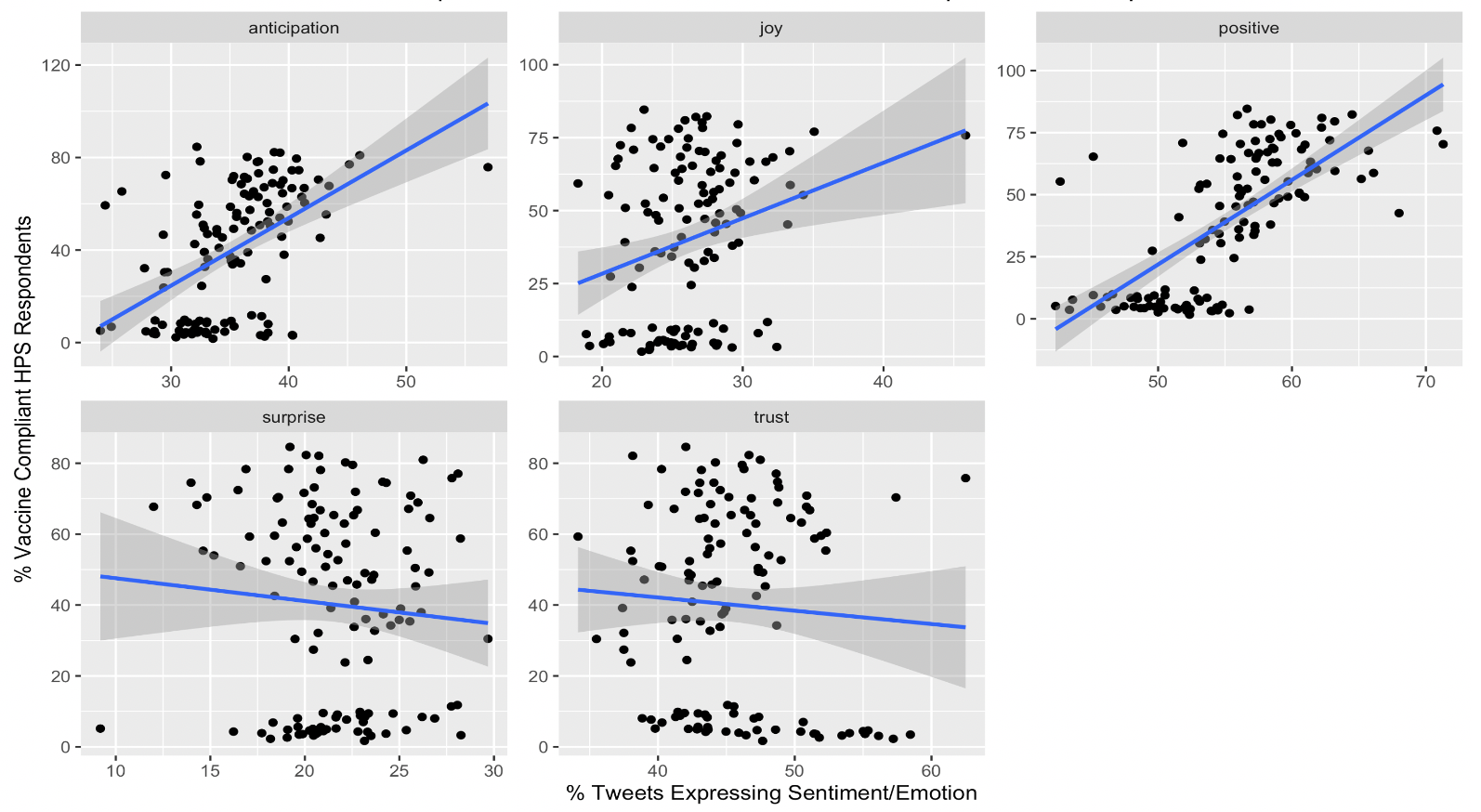


**Figure S8. Relationship Between Predictors and % Vaccine Hesitant HPS Respondents** The percentage of tweets expressing negative sentiment, fear, sadness, and anger have a negative relationship with the percent of vaccine hesitant survey respondents. The percentage of tweets expressing anticipation and disgust have a weak positive relationship with the percent of vaccine hesitant survey respondents.


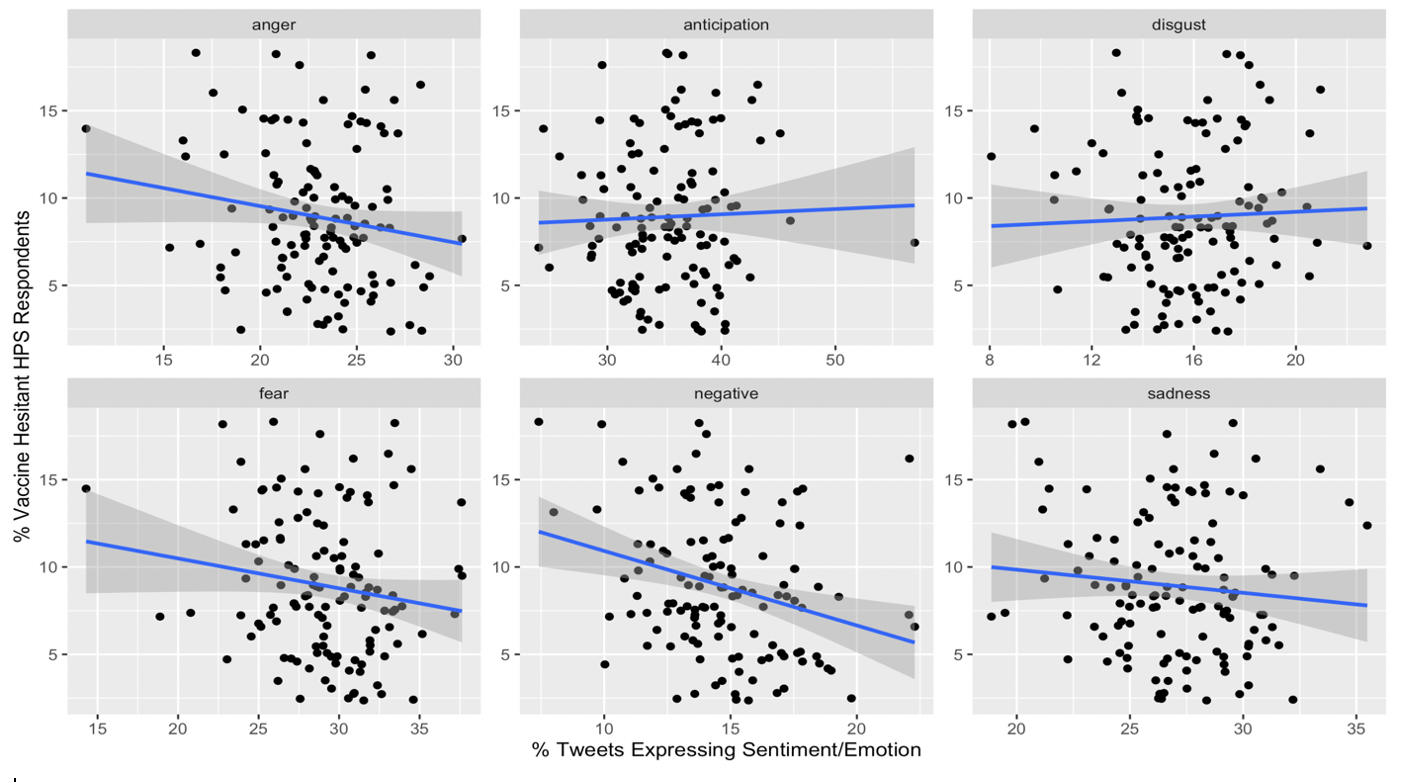

Supplement: Multimedia Appendix 1 [file infodemiology_v3i1e43700_app1.doc]
